# Differential myelinated and unmyelinated sensory and autonomic skin nerve fiber involvement in patients with ophthalmic postherpetic neuralgia

**DOI:** 10.3389/fnana.2015.00105

**Published:** 2015-08-04

**Authors:** Andrea Truini, Maija Haanpaa, Vincenzo Provitera, Antonella Biasiotta, Annamaria Stancanelli, Giuseppe Caporaso, Lucio Santoro, Giorgio Cruccu, Maria Nolano

**Affiliations:** ^1^Department of Neurology and Psychiatry, Sapienza University of RomeRome, Italy; ^2^Department of Neurosurgery, Helsinki University Central HospitalHelsinki, Finland; ^3^Neurology Division “Salvatore Maugeri” Foundation—Institute of Telese Terme (BN) ItalyTelese Terme, Italy; ^4^Department of Neurosciences, Reproductive and Odontostomatological Sciences, University Federico II of NaplesNaples, Italy

**Keywords:** postherpetic neuralgia, skin biopsy, trigeminal nerve, neuropathic pain, intraepidermal nerve fiber

## Abstract

Postherpetic neuralgia (PHN) is a common and exceptionally drug-resistant neuropathic pain condition. In this cross-sectional skin biopsy study, seeking information on the responsible pathophysiological mechanisms we assessed how ophthalmic PHN affects sensory and autonomic skin innervation. We took 2-mm supraorbital punch skin biopsies from the affected and unaffected sides in 10 patients with ophthalmic PHN. Using indirect immunofluorescence and a large panel of antibodies including protein gene product (PGP) 9.5 we quantified epidermal unmyelinated, dermal myelinated and autonomic nerve fibers. Although skin biopsy showed reduced epidermal and dermal myelinated fiber density in specimens from the affected side, the epidermal/dermal myelinated nerve fiber ratio was lower in the affected than in the unaffected side (*p* < 0.001), thus suggesting a predominant epidermal unmyelinated nerve fiber loss. Conversely, autonomic skin innervation was spared. Our study showing that ophthalmic PHN predominantly affects unmyelinated nerve fiber and spares autonomic nerve fiber might help to understand the pathophysiological mechanisms underlying this difficult-to-treat condition.

## Introduction

Ophthalmic postherpetic neuralgia (PHN) is a disabling and extensively investigated neuropathic pain condition caused by herpes zoster and persisting or recurring for at least 3 months in areas supplied by the ophthalmic division of the trigeminal nerve (IHS Classification, 2013).^1^

Studies assessing cutaneous innervation in skin biopsy specimens have provided new insight into the mechanisms underlying neuropathic pain in several conditions (Sommer and Lauria, 2007). Skin biopsy is a minimally invasive diagnostic tool used to assess nerve fiber in the human epidermis and dermis. Epidermal innervation consists mainly of unmyelinated C-fiber terminals, with relatively few small myelinated Aδ-fibers that lose their myelin sheath and reach the epidermis as unmyelinated free nerve endings. Conversely, dermal innervation consists mainly of large myelinated Aβ-fibers that send branches to dermal corpuscles and hair follicles (Nolano et al., 2003, 2013).

Most morphometric studies used bright-field immunohistochemistry with protein gene product (PGP) 9.5 immunostaining and assessed epidermal innervation alone. An approach based on immunofluorescence and confocal microscopy using double and triple immunostaining with an extensive panel of antibodies including the pan-neuronal marker PGP 9.5, the basement membrane marker, collagen IV (ColIV), and antibodies for various myelinated and unmyelinated nerve fiber populations, has proved to provide distinct information on sensory and autonomic nerve fibers, and possibly assess skin innervation more accurately (Doppler et al., 2012; Nolano et al., 2013).

Although previous skin biopsy studies have shown a severe epidermal nerve fiber (ENF) loss in the affected thoracic dermatomes of patients with PHN (Oaklander, 2001; Petersen et al., 2010), they did not directly investigate ophthalmic PHN, nor did they distinguish epidermal unmyelinated and dermal myelinated fibers, or accurately detail autonomic nerve fiber involvement. Having this information might more precisely define the pathophysiological mechanisms underlying PHN, thus possibly improving the way we diagnose and cure this exceptionally drug-resistant neuropathic pain condition.

Continuing our research interest into the mechanisms underlying neuropathic pain in peripheral nerve diseases (Truini et al., 2014) including PHN (Truini et al., 2008), in this clinical and morphometric study, we aimed at investigating how ophthalmic PHN affects myelinated and unmyelinated sensory and autonomic skin nerve fibers. To do so we took 2-mm supraorbital punch skin biopsies from the affected side and unaffected mirror side in ten patients with ophthalmic PHN. We processed the skin specimens with indirect immunofluorescence, examined the specimens by confocal microscopy, distinguished epidermal unmyelinated and dermal myelinated innervation, and described autonomic nerve fiber involvement.

## Materials and Methods

We enrolled 10 patients (3 F, 7 M; 74.5 ± 4.4 years) with ophthalmic PHN in the Department of Neurology, Sapienza University, Rome. Ophthalmic PHN was diagnosed in accordance with the International Headache Society diagnostic criteria: pain persisting or recurring for at least 3 months in the area supplied by the ophthalmic division of the trigeminal nerve, and caused by herpes zoster. Exclusion criteria were cognitive impairment, other central and peripheral nervous system diseases. No patient was treated with topical pain medications (i.e., lidocaine and capsaicin).

The Institutional Review Board of the Department of Neurology, Policlinico Umberto I, Rome, approved the study and patients gave their informed consent.

### Clinical and Neurophysiological Assessment

All participants underwent a general examination and a neurologic examination, with full sensory testing including negative (tactile, pinprick, and thermal hypoesthesia) and positive symptoms (ongoing spontaneous pain, paroxysmal pain) and signs (mechanical dynamic allodynia, and pinprick hyperalgesia). The presence and severity of negative symptoms were assessed by comparing the affected side with the mirror image on the healthy side. The severity of the different pain types (i.e., ongoing spontaneous pain, paroxysmal pain, mechanical dynamic allodynia, pinprick hyperalgesia) was rated on a 0–10 numeric rating scale (NRS; 0 = no sensation, 10 = worst possible pain).

In all patients, we recorded the Aβ-myelinated fiber-mediated blink reflex, with methods adhering to the recommendations of the International Federation of Clinical Neurophysiology guidelines (Deuschl and Eisen, 1999). To investigate Aδ and C fiber function we recorded laser evoked potentials, according to a previously reported technique (Truini et al., 2008).

### Skin Biopsy

**Figure 1:**
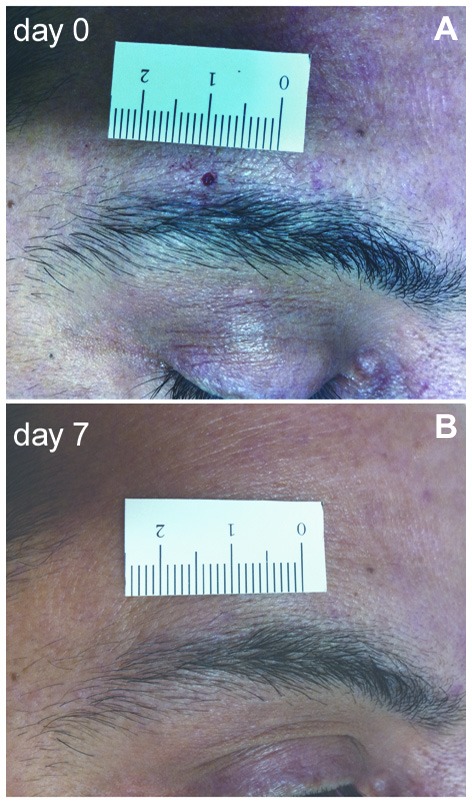
**Two-mm supraorbital punch skin biopsy. (A)** Photograph of a patient with postherpetic neuralgia (PHN) showing the 2-mm skin wound immediately after the punch. **(B)** After 1 week, the scar is hardly visible.

Two-mm punch skin biopsies were taken immediately above the eyebrow from the affected and unaffected mirror side. The wound healed in a few days without a visible scar (Figure 1). Skin samples were processed with immunohistochemical techniques, as previously described (Nolano et al., 2013). Specimens were fixed overnight in Zamboni solution, cryoprotected in 20% sucrose in phosphate-buffered saline and cut into 50-μm-thick sections on a freezing microtome (Leica 2000R, Germany). Free-floating sections were processed for indirect immunofluorescence using an antibody panel to stain sensory, autonomic skin innervation and vascular structures, including PGP9.5, calcitonin gene-related peptide (CGRP), substance P (SubP) and vasoactive intestinal peptide (VIP) antibodies (Table 1). Digital images were acquired using non-laser confocal microscopy (CARV confocal system; ATTO Biosciences, USA and Apotome confocal system; Zeiss, Germany).

**Table 1:** **Abbreviation, source and dilution of immunohistochemical markers**.

Marker	Abbreviation	Source	Dilution	Targeted structure
Rabbit anti protein gene product 9.5	r-PGP	SpringBio	1:400	Pan-neuronal
Rabbit anti vasoactive intestinal peptide	r-VIP	Immunostar	1:1000	Cholinergic fibers
Mouse anti vasoactive intestinal peptide	m-VIP	Santa Cruz	1:300	Cholinergic fibers
Mouse anti protein gene product 9.5	m-PGP	AbDSerotec	1:800	Pan-neuronal
Mouse anti collagen IV	m-Col IV	Chemicon	1:800	Basal membrane, vessels
Rabbit anti substance p	r-Sub P	Incstar	1:1000	Nociceptive fibers
Rabbit anti calcitonin gene related peptide	r-CGRP	Amersham	1:1000	Nociceptive fibers
Rabbit anti s100	r-s100	Maxim Biotech	1:10	Schwann cells
Mouse	m-MBP	Ultraclone	1:800	Myelinated fibers
Rabbit anti dopamine β hydroxilase	r-DβH	Chemicon	1:150	Noradrenergic fibers
ULEX europaeus agglutinin 1	ULEX	Vector	1:100	Epithelia and endothelia

ENF density was assessed in the 2-mm skin punch biopsy samples by applying Neurolucida software (MicroBrightField Bioscience, USA) to four 20× z-series confocal images (2 μm × 16 increments), obtained from four randomly-selected sections for each sample. Dermal myelinated nerve fibers were quantified using anti-myelin basic protein antibodies in the entire dermal surface including 1 mm in depth from the basement membrane in three random sections for each sample, following previously published procedure (Nolano et al., 2013). A calibrated grid (2 × 1 mm) with a series of seven equidistant parallel lines was applied on a digital non-confocal 5× original magnification image of myelin basic protein-stained sections. All intercepts between myelinated fibers and the grid lines were counted. Dermal myelinated nerve fiber density was calculated as the number of fibers intercepting the grid per dermal area (intercepts per mm^2^). A single operator blindly quantified skin nerve fiber in each sample. VIP-ir sudomotor nerve length density (nm/mm^3^) was calculated using the nerve tracing module Autoneuron, a part of Neurolucida software [Microbrightfield Bioscience (MBFB), Williston, VT, USA], as previously described (Provitera et al., 2014). To assess peptidergic fibers (SubP, CGRP and VIP-ir) in the subepidermal neural plexus, within 100 μm from the basement membrane, a semiquantitative scoring was applied (0 = absence of fibers, 1 = rare fiber segments, shorter than 10 μm, 2 = few fibers, populating largely denervated dermis, 3 = normal, several fibers covering most of the subepidermal neural plexus).

Skin biopsy findings in the 10 patients were compared with a matched sample of 10 healthy subjects (epidermal nerve fiber density: 18.9 ± 3.7/mm, dermal myelinated fiber density: 6.7 ± 1.4/mm; Nolano et al., 2013).

### Statistical Analysis

All data are expressed as mean ± SD. Normal distribution of epidermal and dermal myelinated fiber density was evaluated using D’Agostino and Pearson omnibus normality test. The Wilcoxon matched-pair test was used for comparing neurophysiological and skin biopsy variables between unaffected and affected sides. The Mann Whitney test was used for comparing skin biopsy variables between PHN patients and normal controls. Correlations between the ENF density and PHN duration were calculated with the Pearson’s *r* correlation coefficient. *P* values less than or equal to 0.05 were considered to indicate statistical significance.

## Results

At clinical examination all ten patients complained of sensory deficits involving all sensory modalities and reported ongoing spontaneous pain (NRS: 4.3 ± 1.4); five patients also complained of mechanical dynamic allodynia (NRS: 3.5 ± 0.7).

Neurophysiologic testing showed severe abnormalities involving all variables tested. In all patients Aβ-fiber-mediated blink reflex, stimulating the affected side, elicited abnormal (delayed or absent) responses. Laser perceptive thresholds were higher and Aδ- and C-laser evoked potential amplitudes lower after stimulating the affected side than after stimulating the unaffected side (*p* < 0.05, Wilcoxon test). In 2 of the 10 patients, laser evoked potentials related to C-fiber stimulation were absent also in the unaffected side (Table 2).

**Table 2:** **Abbreviation, source and dilution of immunohistochemical markers**.

	Normal side (mean ± SD)	Affected side (mean ± SD)	*p*
R1 latency (ms)	11.3 ± 0.6	14.4 ± 1.4*	0.03
Pinprick laser perceptive threshold (mJ/mm^2^)	58.5 ± 12.1	101.6 ± 43.3	0.004
Warm laser perceptive threshold (mJ/mm^2^)	26.8 ± 10.9	53.20 ± 13.2	0.002
Aδ-fiber laser evoked potential amplitude (μV)	18.3 ± 4.9	6.1 ± 7.2**	0.006
C-fiber laser evoked potential amplitude (μV)	10.5 ± 8.6***	3.1 ± 4.3****	0.02
Epidermal nerve fiber density/mm	17.2 ± 8.9	3.1 ± 3.3	0.002
Myelinated dermal nerve fiber density/mm	6.6 ± 5.0	2.1 ± 2.8	0.004
Epidermal/dermal nerve fiber density ratio	4.3 ± 5.3	1.1 ± 1.0	0.006

*Absent in 4 patients; **absent in 5 patients; ***absent in 2 subjects; ****absent in 6 subjects.

**Figure 2:**
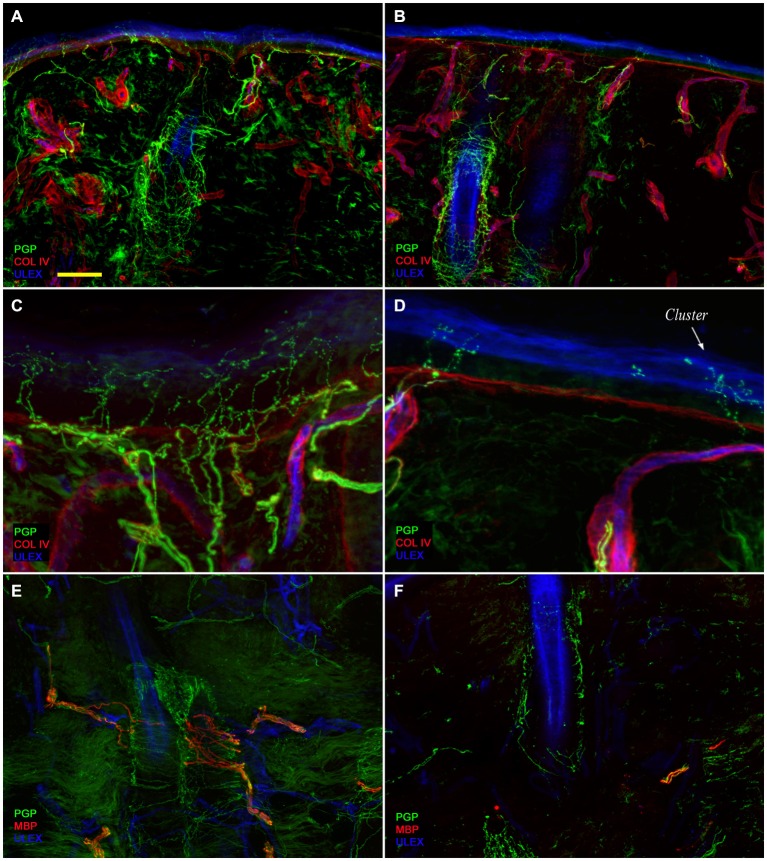
**Skin innervation**. Confocal images showing skin innervation in the unaffected **(A,C,E)** and affected side **(B,D,F)** in a representative patient with PHN. Severe epidermal and dermal nerve fiber loss is visible in skin biopsy specimens from the affected side (**B** vs. **A**). The epidermis in the affected side displays a long tract devoid of nerve fibers and nerve fiber clusters (**D** vs. **C**). Striking unmyelinated and myelinated nerve fiber loss around hair follicles (**F** vs. **E**). Bars: 100 μm in **(A,B,E,F)**; 25 μm in **(C,D)**.

**Figure 3:**
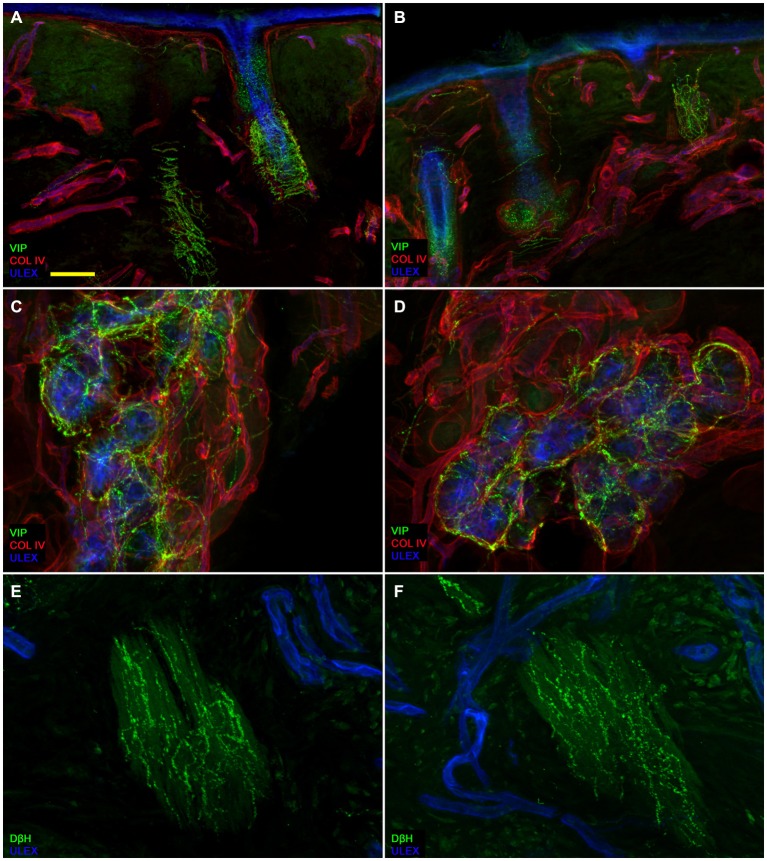
**Autonomic skin innervation**. Confocal images showing Autonomic innervation to dermal annexes in the affected **(A,C,E)** and unaffected side **(B,D,F)**. The upper dermis around hair follicles in the affected side contains fewer vasoactive intestinal peptide-immunoreactive (VIP-IR) fibers than the same dermal area in the unaffected side (**A** vs. **B**) whereas VIP-ir sudomotor and DbH-ir pilomotor innervation is well endowed (**D** and **F** vs. **C** and **E**). Bars: 100 μm in **(A,B)**; 50 μm in **(C,D)**, 25 μm in **(E,F)**.

Confocal immunofluorescence analysis showed that skin innervation in the supraorbital punch skin biopsies from patients with PHN differed strikingly between the unaffected (Figures 2A,C,E) and affected sides (Figures 2B,D,F). Whereas skin biopsy samples from the unaffected side displayed a fairly dense and regular innervation, the epidermis in the affected side appeared poorly and unevenly innervated with clusters and long tracts devoid of nerve fibers (Figures 2A–D). Semiquantitative analysis showed that the subepidermal plexus appeared poor and deranged and, compared to the unaffected side, contained less (*p* < 0.001) CGRP (1.4 ± 0.8 vs. 2.5 ± 0.5), SubP (1.1 ± 0.6 vs. 2.3 ± 0.7), and VIP (1.0 ± 0.7 vs. 2.2 ± 0.8) immunoreactive (IR) nerve fibers. Hair follicles appeared poorly innervated with few unmyelinated and myelinated fibers (Figures 2F,E). Epidermal and dermal myelinated fiber loss varied from moderate to severe and 3 of the 10 patients had complete skin denervation. The few surviving epidermal axons were clustered between areas of denervated epidermis. Myelinated fibers showed several morphological abnormalities, such as fragmentation, frequent occurrence of longer nodal length than normal (7 μm or longer; Nolano et al., 2003), and caliber irregularities with enlargement and shrinkage (change in diameter over 100%) within the same internode. These features were rarely observed in the unaffected side. In contrast with sensory innervation, both components, adrenergic and cholinergic, of autonomic innervation to the dermal annexes appeared relatively preserved (Figure 3). The unaffected and affected sides displayed numerous cholinergic (VIP-ir) sudomotor (Figures 3C,D) and adrenergic (DbH-ir) pilomotor (Figures 3E,F) fibers. Cholinergic sudomotor nerve fiber density did not differ between affected (1.9 ± 0.7 nm/mm^3^) and unaffected side (2.0 ± 0.5 nm/mm^3^).

Quantitative analysis (based on analysis of PGP9.5 and myelin basic protein (MBP) immunoreactivity) of the skin biopsy samples showed a significantly lower epidermal and dermal myelinated fiber density in the affected side than in the unaffected side (3.1 ± 3.3 and 2.1 ± 2.8 vs. 17.2 ± 8.9 and 6.6 ± 5.0/mm^2^; *p* < 0.01, Table 2). The ratio between epidermal and dermal myelinated nerve fibers was significantly lower in the affected than in the unaffected side (1.1 ± 1.0 vs. 4.3 ± 5.3; *p* < 0.01).

**Figure 4:**
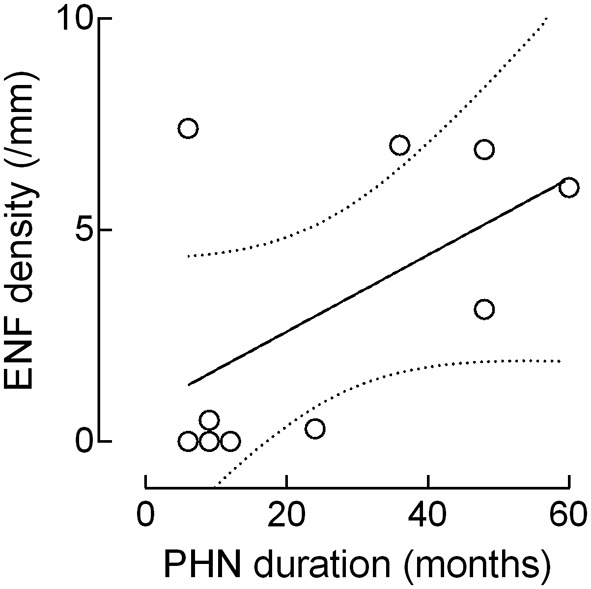
**Correlation between epidermal nerve fiber (ENF) density and PHN duration**. Although the ENF density increased with the PHN duration, this correlation missed the statistical significance (epidermal nerve fiber: *r*: 0.5574, *p* = 0.09).

Although the ENF density increased with the PHN duration, this correlation failed to reach statistical significance (*r*: 0.5574, *p* = 0.09; Figure 4).

The mean values for unmyelinated and myelinated nerve-fiber densities in the unaffected side did not differ from controls (epidermal nerve fiber density: 17.2 ± 8.9/mm vs. 18.9 ± 3.7/mm; dermal myelinated fiber density: 6.6 ± 5.0/mm vs. 6.7 ± 1.4/mm; *p* > 0.3, by Mann-Whitney test). However, in the two patients with abnormal C-fiber-mediated laser evoked potentials, skin biopsy showed an epidermal and a dermal myelinated nerve fiber loss also in the unaffected side (0.8 and 3.6/mm in the unaffected and 0.0 and 0.3/mm in the affected side).

## Discussion

Our clinical and morphometric study, assessing skin innervation in supraorbital punch skin biopsies with indirect immunofluorescence and confocal microscopy, provides new information specifying that although ophthalmic PHN affects both epidermal unmyelinated and dermal myelinated nerve fibers, it predominantly damages unmyelinated nerve fibers and spares autonomic skin innervation.

Clinical examination showed that all patients had tactile, pinprick, and warm hypoesthesia. Accordingly, all the three neurophysiological variables we recorded (the Aβ-fiber mediated blink reflex, Aδ- and C-laser evoked potentials) were abnormal. Whereas clinical assessment and neurophysiologic testing showed both myelinated and unmyelinated fiber damage, skin biopsy assessment specified that PHN-related damage to skin innervation differed in myelinated and unmyelinated fibers. More specifically, the lower unmyelinated/myelinated ratio in the affected side than in the unaffected mirror side suggests a predominant epidermal unmyelinated nerve fiber loss. This new morphometric finding agrees with previous studies that investigated epidermal innervation alone and, showed a variable unmyelinated fiber loss (Oaklander, 2001; Petersen et al., 2010; Reda et al., 2013). The predominant unmyelinated nerve fiber involvement we describe in patients with PHN fits in with their typically ongoing pain. This type of pain probably reflects secondary neuroplastic changes and spontaneous hyperactivity in the anatomically denervated second-order nociceptive neurons (Truini et al., 2013). Owing to the small study sample we could not reliably investigate possible correlations between pain and clinical, neurophysiological and morphological abnormalities. This question deserves investigation in a further study enrolling more patients.

In this skin biopsy study, using indirect immunofluorescence and confocal microscopy, we provide previously unreported detailed information on skin autonomic innervation in patients with ophthalmic PHN. Whereas autonomic innervation to sweat glands and to erector pilorum muscles was spared, we observed a poor representation of VIP-IR fibers in the upper dermis, around vessels and hair follicles, where there was also a poor representation of CGRP and SubP. Some of these fibers modulate hair follicle function and facial vasomotor control, and may originate from the sensory trigeminal nerve (Izumi, 1995, 1999). This origin may explain their involvement in PHN whereas the uninvolved sudomotor and pilomotor VIP-IR nerve fibers probably originate from the superior cervical sympathetic ganglia and from cranial parasympathetic nerves that PHN leaves intact. Given that many studies suggest that the autonomic nervous system might participate in the development of pain (Fields et al., 1998), the autonomic nerve fiber sparing we describe here might support the proposed indirect coupling between intact sympathetic postganglionic and damaged primary afferent unmyelinated neurons (Drummond et al., 2014).

In our patients with ophthalmic PHN skin biopsy findings showed that the few surviving axons in the affected side were clustered between areas of denervated epidermis and that ENF density tended to increase with disease duration. This observation leads us to conjecture that the skin could undergo some nerve remodeling over time. These changes could take place when the surviving axons sprout collaterals in an attempt to occupy the denervated territory. This anatomical remodeling may also bring about some functional improvement. Admittedly, our hypothesis suggesting a partial reinnervation over time in patients with ophthalmic PHN argues against pivotal studies addressing the natural history of thoracic PHN (Petersen et al., 2010; Reda et al., 2013). These studies using functional measures and skin biopsy conclude that even at 7.7 years, there is only a modest recovery of sensory function and no anatomic recovery despite pain resolution in the majority of patients. In particular, the study by Reda and colleagues showed that although pain and most measures of sensory function, sensory symptoms, and capsaicin response continued a trend toward improvement during a long-term follow-up, many subjects still experienced sensory abnormalities. Although we cannot exclude that the reliability of our findings might be influenced by the small sample of patients, whether the the trigeminal system, a peculiar territory, is more predisposed to skin reinnervation than other body areas remains an interesting question for future research.

The mean epidermal and dermal myelinated fiber density and their ratio in the unaffected mirror side did not differ from controls. However in two of the ten patients we studied, C-fiber-related laser evoked potentials were absent after stimulating the unaffected side and both patients had mild epidermal fiber loss, though less severe in the unaffected than in the affected side. This finding is in line with previous clinical, neurophysiological and skin biopsy studies showing bilateral nerve fiber damage and supports the hypothesis that PHN may occasionally cause mild, subclinical sensory afferent pathway involvement in the unaffected side (Haanpää et al., 1997; Oaklander et al., 1998; Truini et al., 2008).

Our skin biopsy study showing that ophthalmic PHN differentially affects epidermal and dermal myelinated fibers and spares the autonomic skin innervation provides new information for understanding the mechanisms underlying this disabling condition.

## Author Contributions

AT, MN: Study concept and design; AT, AB, VP, AS, GC, MN: acquisition, analysis and interpretation of data; AT, MN: drafting of the manuscript; MH, GC, LS, MN: critical revision 3 of the manuscript for important intellectual content.

## Conflict of Interest Statement

The authors declare that the research was conducted in the absence of any commercial or financial relationships that could be construed as a potential conflict of interest.

## Footnotes

^1^http://www.ihs-classification.org/_downloads/mixed/International-Headache-Classification-III-ICHD-III-2013-Beta.pdf

## References

[B1] Deuschl G. Eisen A. (1999). Long-latency reflexes following electrical nerve stimulation. The international federation of clinical neurophysiology. Electroencephalogr. Clin. Neurophysiol. Suppl. 52, 263–268. 10590995

[B2] Doppler K. Werner C. Henneges C. Sommer C. (2012). Analysis of myelinated fibers in human skin biopsies of patients with neuropathies. J. Neurol. 259, 1879–1887. 10.1007/s00415-012-6432-7 22310941

[B3] Drummond E. S. Dawson L. F. Finch P. M. Bennett G. J. Drummond P. D. (2014). Increased expression of cutaneous α1-adrenoceptors after chronic constriction injury in rats. J. Pain 15, 188–196. 10.1016/j.jpain.2013.10.010 24212069

[B4] Fields H. L. Rowbotham M. Baron R. (1998). Postherpetic neuralgia: irritable nociceptors and deafferentation. Neurobiol. Dis. 5, 209–227. 10.1006/nbdi.1998.0204 9848092

[B5] Haanpää M. Häkkinen V. Nurmikko T. (1997). Motor involvement in acute herpes zoster. Muscle Nerve 20, 1433–1438. 10.1002/(sici)1097-4598(199711)20:11<1433::aid-mus11>3.0.co;2-2 9342160

[B6] IHS Classification (2013). The international classification of headache disorders, 3rd edition (beta version). http://www.ihs-classification.org/_downloads/mixed/International-Headache-Classification-III-ICHD-III-2013-Beta.pdf 10.1177/0333102413485658 23771276

[B7] Izumi H. (1995). Reflex parasympathetic vasodilatation in facial skin. Gen. Pharmacol. 26, 237–244. 10.1016/0306-3623(94)00155-g 7590072

[B8] Izumi H. (1999). Nervous control of blood flow in the orofacial region. Pharmacol. Ther. 81, 141–161. 10.1016/s0163-7258(98)00040-0 10190584

[B9] Nolano M. Provitera V. Caporaso G. Stancanelli A. Leandri M. Biasiotta A.. (2013). Cutaneous innervation of the human face as assessed by skin biopsy. J. Anat. 222, 161–169. 10.1111/joa.12001 23078075 PMC3632221

[B10] Nolano M. Provitera V. Crisci C. Stancanelli A. Wendelschafer-Crabb G. Kennedy W. R.. (2003). Quantification of myelinated endings and mechanoreceptors in human digital skin. Ann. Neurol. 54, 197–205. 10.1002/ana.10615 12891672

[B11] Oaklander A. L. (2001). The density of remaining nerve endings in human skin with and without postherpetic neuralgia after shingles. Pain 92, 139–145. 10.1016/s0304-3959(00)00481-4 11323135

[B12] Oaklander A. L. Romans K. Horasek S. Stocks A. Hauer P. Meyer R. A. (1998). Unilateral postherpetic neuralgia is associated with bilateral sensory neuron damage. Ann. Neurol. 44, 789–795. 10.1002/ana.410440513 9818935

[B13] Petersen K. L. Rice F. L. Farhadi M. Reda H. Rowbotham M. C. (2010). Natural history of cutaneous innervation following herpes zoster. Pain 150, 75–82. 10.1016/j.pain.2010.04.002 20457490

[B14] Provitera V. Nolano M. Caporaso G. Stancanelli A. Manganelli F. Iodice R.. (2014). Postganglionic sudomotor denervation in patients with multiple system atrophy. Neurology 82, 2223–2239. 10.1212/WNL.0000000000000518 24838791

[B15] Reda H. Greene K. Rice F. L. Rowbotham M. C. Petersen K. L. (2013). Natural history of herpes zoster: late follow-up of 3.9 years (n=43) and 7.7 years (n=10). Pain 154, 2227–2233. 10.1016/j.pain.2013.04.015 23719573

[B16] Sommer C. Lauria G. (2007). Skin biopsy in the management of peripheral neuropathy. Lancet Neurol. 6, 632–642. 10.1016/S1474-4422(07)70172-2 17582363

[B17] Truini A. Biasiotta A. Di Stefano G. Leone C. La Cesa S. Galosi E.. (2014). Does the epidermal nerve fibre density measured by skin biopsy in patients with peripheral neuropathies correlate with neuropathic pain? Pain 155, 828–832. 10.1016/j.pain.2014.01.022 24486884

[B18] Truini A. Galeotti F. Haanpaa M. Zucchi R. Albanesi A. Biasiotta A.. (2008). Pathophysiology of pain in postherpetic neuralgia: a clinical and neurophysiological study. Pain 140, 405–410. 10.1016/j.pain.2008.08.018 18954941

[B19] Truini A. Garcia-Larrea L. Cruccu G. (2013). Reappraising neuropathic pain in humans–how symptoms help disclose mechanisms. Nat. Rev. Neurol. 9, 572–582. 10.1038/nrneurol.2013.180 24018479

